# Perturbed Inflammatory, Proliferation, Regulatory, And Neurodegeneration Signaling Pathways Are Associated With Trait Anxiety In Cancer Survivors

**DOI:** 10.21203/rs.3.rs-9383360/v1

**Published:** 2026-07-17

**Authors:** Nidhi Thati, Julia Trudeau, Ding Quan Ng, Esther Chavez-Iglesias, Anand Dhruva, Adam Olshen, Raymond Chan, Alexandre Chan, Kord M. Kober

**Affiliations:** University of California, San Francisco; University of California, Irvine; University of California, Irvine; University of California, San Francisco; University of California, San Francisco; University of California, San Francisco; Flinders University; University of California, Irvine; University of California, San Francisco

**Keywords:** cancer, trait anxiety, transcriptomics, survivors

## Abstract

**Introduction:**

Trait anxiety is a prevalent yet under-studied symptom in cancer survivors, linked to stress and reduced quality of life. The purpose of this study was to evaluate perturbed biological pathways associated with trait anxiety severity in both cancer survivors and the general population to understand overlap between these cohorts.

**Methods:**

Participants in the Multi-Ethnic Study of Atherosclerosis (MESA), a prospective longitudinal study, were used in this analysis (PHD #39341). Complete gene expression data were available for 1,093 participants at the first visit, and trait anxiety was assessed using ten questions from the Spielberger Trait Anxiety Scale. Participants were split into low (< 3rd quartile) and high (≥ 3rd quartile) trait anxiety groups. Analyses were performed separately for cancer survivors and the general population. Differential gene expression was evaluated between low and high trait anxiety groups, adjusting for covariates. Pathway impact analysis was used to identify perturbed pathways (FDR < 0.025).

**Results:**

Forty-two pathways were perturbed in the cancer survivor cohort (n = 74, low anxiety = 63, high anxiety = 11). Eighty pathways were perturbed in the non-cancer cohort (n = 222, low anxiety = 181, high anxiety = 41). Thirty-six pathways overlapped between cohorts, including inflammatory, proliferation, neurodegeneration, and regulatory pathways. Six pathways were unique to cancer survivors. Inflammatory pathways emerged as a shared mechanism across both cohorts.

**Conclusions:**

This study is the first to evaluate transcriptomic perturbations in pathways associated with trait anxiety in cancer survivors and a matched non-cancer cohort. Findings identify shared and distinct mechanisms underlying trait anxiety in cancer survivors, suggesting potential targets for future interventions.

## INTRODUCTION

Trait anxiety is a common psychosocial symptom experienced by cancer survivors, reflecting a stable and enduring tendency to experience heightened anxiety levels across contexts[1, 2]. Unlike state anxiety, which is situational, trait anxiety represents a chronic personality characteristic that can predispose individuals to the development of Generalized Anxiety Disorder (GAD)[1]. Cancer survivors may have up to a 2.7-fold increased risk of developing anxiety[3], and both state and trait anxiety can negatively impact their daily quality of life[4]. This heightened risk could be due to increased self-reported cognitive symptoms and depression in cancer survivors[4], contributing to the pathophysiology of trait anxiety. However, little is known about the biological mechanisms contributing to trait anxiety or how these mechanisms differ in cancer survivors as compared to the general population. Understanding biological mechanisms underlying trait anxiety and how they are both common and distinct between cancer survivors and the general population is essential for developing targeted interventions.

Previous neurobiological work shows that both anxiety and stress responses engage overlapping brain areas, including the hypothalamus and amygdala, suggesting that several pathways implicated in trait anxiety are also tied to stress[2]. In cancer survivors, specifically, anxiety has been linked to chronic stress levels and dysregulation of the hypothalamic-pituitary-adrenal (HPA) axis[5].

Studies evaluating for the biological mechanisms of anxiety have focused on the non-cancer population and primarily on the role of inflammatory and immune processes, particularly in the symptomology of GAD[6–8]. In addition to inflammatory markers, stress-induced anxiety is associated with inflammation and dysfunction of the blood-brain barrier[9], and genetic variations in genes within the HPA axis are associated with self-assessed anxiety in healthy individuals[10]. Despite these insights, current research has focused primarily on specific genes and signaling modulators rather than pathway-level perturbations.

By examining the entire transcriptome or genome, rather than focusing on specific biomarkers and predefined pathways, data-driven analyses can reveal additional mechanisms and refine current ones. Certain previously hypothesized mechanisms come from a recent preclinical study that identified gene clusters associated with trait anxiety involved in cellular localization, cellular transport, potassium signaling, and transcription[11]. Immune signaling pathways have also been implicated in anxiety symptoms among cancer survivors[12], as well as neurodegenerative pathways that link neuroinflammation and trait anxiety severity[13]. Certain regulatory pathways, including shaping of the extracellular matrix, have also been previously associated with trait anxiety[11]. Although numerous studies have evaluated for molecular mechanisms associated with state anxiety in oncology patients[7, 14, 15], no studies were identified that have evaluated for trait anxiety in cancer survivors.

The purposes of this study were (1) to evaluate for perturbed biological pathways associated with trait anxiety severity in cancer survivors, (2) evaluate for perturbed biological pathways associated with trait anxiety severity in the general population, and (3) identify common and distinct perturbed pathways between these cohorts. We hypothesize that there will be both common and distinct mechanisms that underlie trait anxiety in cancer survivors and the general population. Cancer survivors may benefit from a broader understanding of the mechanisms underlying trait anxiety. If substantial overlap exists with the general population, these findings may inform our understanding of trait anxiety in cancer survivors. The distinct perturbations specific to cancer survivors may reflect cancer-specific experiences or unique biological alterations, suggesting opportunities for more targeted interventions.

## METHODS

### Participants and settings

**Figure 1:**
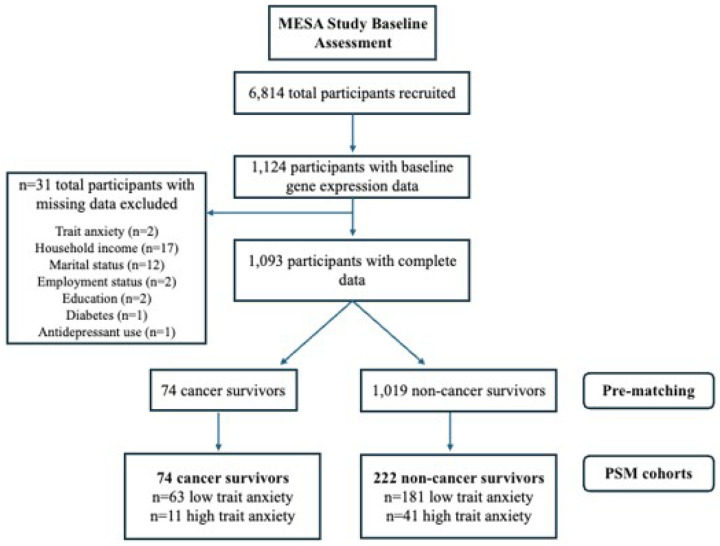
Flowchart of participants in the MESA study and our study.

Participants in the Multi-Ethnic Study of Atherosclerosis (MESA), a prospective longitudinal study, were included in this analysis[16]. The MESA study was designed to recruit participants free of known cardiovascular disease across the strata of sex, race/ethnicity, and age. Participants were recruited across six geographically diverse U.S. field centers. Detailed information on socioeconomic, psychosocial, environmental, and molecular measures was collected. The present analysis is based on 1,093 participants for whom gene expression data, trait anxiety assessments, and covariate data were available from the baseline assessment at Exam 1 (2000–2002) (Fig. 1). Briefly, eligible MESA participants are between the ages of 45 and 84 at enumeration, are African-American, Chinese-American, Caucasian, or Hispanic, and do not meet any of the exclusion criteria. Exclusion criteria include active treatment for cancer and serious medical conditions. Cancer survivors are defined as those with a self-reported history of a cancer diagnosis (i.e., “Has a doctor ever told you that you had any of the following: Cancer”)[17]. Data from the MESA study were obtained through an approved study project (39341) and dbGaP studies phs000209.v13.p3 (Multi-Ethnic Study of Atherosclerosis (MESA) Cohort) and phs001416.v4.p1 (NHLBI TOPMed MESA and MESA Family AA-CAC).

### Instruments

Participants completed a series of questionnaires (e.g., medical history, psycho-social) and assessments. A trait anxiety score was provided based on the assessment at Exam 1 using responses to ten statements from the Trait Anxiety Scale of the Spielberger State-Trait Anxiety Inventory (Supplementary Table 1). Statements were rated from 1 to 4 and summed together. Across all participants in the study with trait anxiety assessed at Exam 1 (n = 6,429), the trait anxiety summary score ranged from 10.00 to 37.00 (median = 15.87, 3rd quartile = 19.00). Given that a previously published cutoff is not available for clinically meaningful levels of high trait anxiety with this set of statements, for this study, the participants were split into low (< 3rd quartile) and high (≥ 3rd quartile) trait anxiety groups. Gene expression was assessed using RNA isolated from peripheral blood mononuclear cells (PBMCs) and quantified as expected counts[18].

### Data analysis

#### Patient demographic, clinical, and psychosocial data

Data from the cancer and non-cancer cohorts were analyzed separately using R (version 4.1, https://www.R-project.org/). Complete case analysis was performed. For each sample, differences in demographic and clinical characteristics between the trait anxiety groups were evaluated using parametric and non-parametric tests. Significant characteristics (p-value of < .05) were included as covariates in the gene expression analyses.

##### Cancer survivor and matched non-cancer cohorts.

To evaluate the generalizability of the findings in the survivor cohort, we created a cohort from participants who did not report previous cancer diagnoses matched in sociodemographic characteristics to the cancer survivors using propensity-score matching (PSM) (i.e., the “PS matched non-cancer cohort”). We used 1:3 matching on propensity scores using the optimal pair-matching algorithm[19]. Variables considered included age, race/ethnicity, gender, employment status, household income, body mass index (BMI), education, and marital status. Briefly, we performed univariate logistic regression analysis between cancer survivors and non-cancer survivor groups and used variables with p < 0.1 for propensity score calculation[20]. Successful matching was defined as all covariates having standardized mean differences of <|0.1|[21]. Post-matching univariate logistic regression analyses were performed to ensure variables not included remained balanced after matching. Matching was completed with R package MatchIt[22].

### Differential gene expression

Differential gene expression was evaluated between low and high trait anxiety groups consistent with our previous studies[15, 23–25]. Briefly, differential expression was quantified using empirical Bayes models using edgeR[26]. These analyses were adjusted for phenotypic characteristics that differed between the low and high anxiety groups in the respective cohorts identified by the univariate tests. The models included surrogate variables not associated with trait anxiety to adjust for variation due to unmeasured sources[27].

### Pathway impact analysis (PIA)

PIA was used to interpret the gene expression results in the context of trait anxiety-related mechanisms[26]. PIA included the results of the differential expression analyses for all genes (i.e., cutoff-free) to determine the probability of pathway perturbations using Pathway Express[28]. A total of 149 signaling pathways were identified using the Kyoto Encyclopedia of Genes and Genomes (KEGG) database (Release 73.0+/01–03, Jan 15)[29]. For both the cancer and non-cancer cohorts, a separate test was performed for each pathway. The significance of the transcriptome-wide PIA was assessed using a strict false discovery rate (FDR) of 0.025 under the Benjamini-Hochberg procedure[30]. These results were evaluated for common and distinct pathways associated with trait anxiety across the cancer survivor and propensity score-matched non-cancer cohort.

## RESULTS

### Participant characteristics

1,093 participants with complete data were eligible for analysis (Fig. 1). A total of 74 cancer survivors were identified (n = 63, low trait anxiety; n = 11, high trait anxiety). Cancer survivors had a median age of 68.5 (IQR: 10), were majority non-Hispanic White (70%) and 51% male, and most had other, multiple, or unspecified cancer diagnoses (43%). The most common distinct diagnoses were breast cancer, colon cancer, non-melanoma skin cancer, and prostate cancer. Survivors with high trait anxiety were more likely to be female (Table 1).

**Figure 2:**
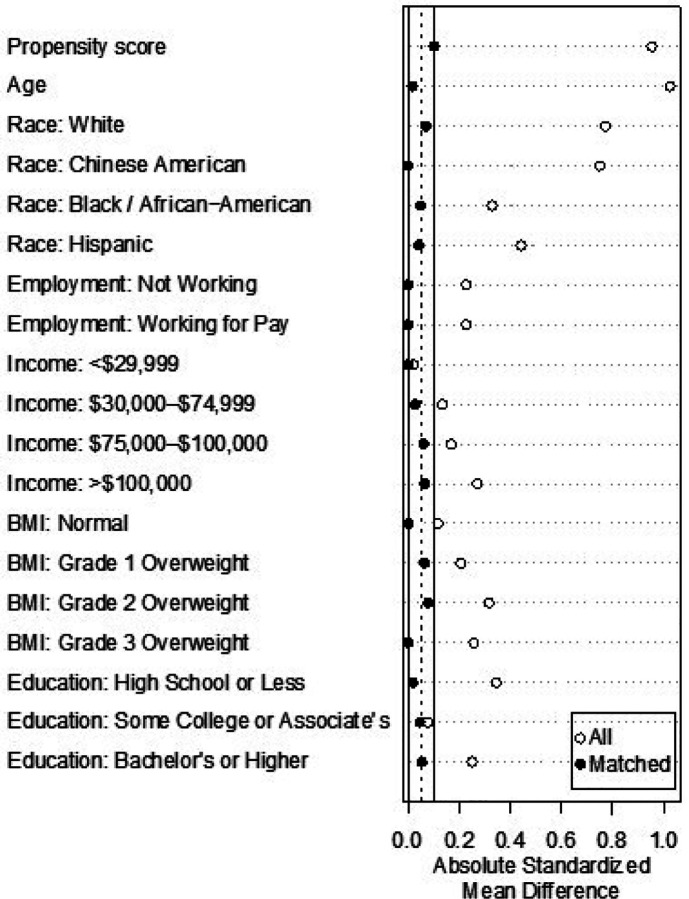
Love plot depicting absolute standardized mean differences for covariates between the entire participant cohort and the propensity score matched cohort.

From 1,019 total non-cancer participants, 222 non-cancer participants were identified for the propensity-score matched cohort (n = 181, low trait anxiety; n = 41, high trait anxiety). After matching, all sociodemographic characteristics were balanced (Fig. 2, Supplemental Table 2). PSM-matched non-cancer participants with high trait anxiety were less likely to be married (Table 2) (p < 0.01). No differences were found in either cohort between low and high trait anxiety and age or self-reported ethnicity. In terms of comorbidities and medication use, no differences were found in either cohort between low and high trait anxiety groups and tricyclic anti-depressant use, a diagnosis of diabetes, or a diagnosis of hypertension (Supplementary Tables 3 and 4).

### Pathway perturbation

Of the 149 signaling pathways evaluated with sufficient data, a total of 42 pathways were significantly perturbed between high and low trait anxiety groups among cancer survivors (Table 3, Supplemental Table 5, Supplemental File 1). A total of 80 pathways were significantly perturbed between high and low trait anxiety groups among the PS-matched non-cancer cohort (Table 4, Supplemental Table 5, Supplemental File 2). Thirty-six perturbed pathways were common between the cancer and non-cancer cohorts. Perturbed pathways were related to the four molecular mechanisms of inflammation & immune signaling, cellular growth and proliferation, neurodegeneration and neurotransmission, and cell structure & regulation. In addition, pathways were identified across both cohorts related to host-pathogen interaction and innate immunity (Supplemental Table 5).

## DISCUSSION

This is the first study to evaluate for perturbed pathways associated with trait anxiety in cancer survivors and across the general population. Our findings suggest that trait anxiety in cancer survivors is associated with inflammation & immune signaling, cellular growth & proliferation, neurodegeneration, and cell structure & regulation. Many of these pathways are concordant with those perturbed in the non-cancer population, suggesting that common and distinct pathways may underlie trait anxiety in cancer survivors.

Many of the common pathways have been identified in previous studies of trait anxiety[7, 31] and in studies of GAD[8] and will not be discussed in detail. The discussion will focus on novel common and distinct pathways within previously hypothesized mechanisms contributing to trait anxiety.

### Demographic and clinical characteristics

Consistent with previous studies[32], within the cancer survivor cohort, female participants were significantly more likely to be in the high anxiety group than males. Anxiety disorders are hypothesized to be more prevalent and disabling in women than men[33].

In the propensity-score matched non-cancer cohort, marital status was significantly different between low and high trait anxiety groups, with matched participants in the high anxiety group being less likely to be married. This is concordant with previous studies that have found that marital status is associated with trait anxiety[34]. Unlike previous studies which have found younger age[35], lower SES[36], race and ethnicity[37], and depression[38] to be associated with anxiety, we did not observe any significant differences in these characteristics between high and low trait anxiety groups among cancer survivors.

### Inflammation & Immune Signaling

Immune signaling and inflammatory pathways have been previously hypothesized to contribute to increased trait anxiety. Inflammation, particularly stress-induced neuroinflammation, can enhance the release of pro-inflammatory cytokines, which modulate neural circuits involved in anxiety[12]. Notably, both cancer and anti-cancer treatments can contribute to the release of pro-inflammatory cytokines, and this serves as a previously hypothesized mechanism of neuropsychiatric symptoms among cancer survivors[39, 40]. All the immune signaling pathways identified in the pathway perturbation analysis of survivors were perturbed in both the survivor and general population cohorts.

Our findings are concordant with previous studies that identified a role of immune signaling in trait anxiety in cancer survivors. For example, the cytokine-cytokine receptor interaction pathway has been previously implicated in trait anxiety among oncology patients and their family caregivers[7]. This study also reported that genetic variations in certain inflammatory cytokine genes were associated with higher trait anxiety, suggesting that perturbations in cytokine inflammatory genes could contribute to higher anxiety in both oncology patients and family caregivers. In a broader context, in a study of healthy men free of acute illnesses with GAD and controls, enrichment of differentially expressed genes was found in immune response and immune cell-specific pathways[6]. Taken together, these findings from both healthy and oncology patients emphasize the potential role of immune response pathways in the biology of trait anxiety.

The chemokine signaling pathway, also perturbed in both the general population and cancer survivors, has been associated with anxiety. In a study of women with moderate to severe anxiety during pregnancy, serum levels of most chemokines were significantly increased in those exhibiting higher levels of anxiety[41]. Preclinical evidence also supports the role of chemokine receptors and signaling in modulating anxiety, resulting in increased or decreased anxiety-like behavior in mouse models[42]. Our findings support this differential role of the chemokine signaling pathway in influencing trait anxiety severity, in both cancer survivors and non-cancer participants. Since all perturbed immune signaling pathways were common between the cancer and non-cancer cohorts, immune signaling forms a common mechanism for trait anxiety, and targeting inflammation-specific pathways in therapeutics can address trait anxiety in cancer survivors.

### Cellular Growth, Cycle, & Proliferation

Numerous signaling pathways were related to cellular growth, cycling, and proliferation, most of which were concordant between the general population and the cancer survivor population. These pathways are involved in transcription, cellular localization, and transport, which are mechanisms previously identified in a preclinical model of trait anxiety[11].

The MAPK signaling pathway is primarily involved in growth, proliferation, inflammatory, and stress responses. It was perturbed in both the survivors and general population and has been previously hypothesized to be a central pathway in the pathophysiology of anxiety disorders[43]. Downstream components of the MAPK signaling pathway are targeted by anxiety-related miRNAs[43], and the pathway is being considered as an interventional target in the treatment of Bipolar Disorder through modulating the MAPK cascade[44]. Therefore, it is likely that this signaling pathway modulates trait anxiety severity.

Other signaling pathways common between the populations, including the mTOR pathway, are implicated in stress-regulatory systems[45]. Similarly, the common ErbB signaling and Hippo signaling pathways have been linked to anxiety and stress in preclinical models[46, 47]. Stress physiology is tightly linked to anxiety[12, 48]. Stress is a significant risk factor for anxiety disorders and stress-induced anxiety has been shown to alter biological pathways similar to innate anxiety, including immune signaling pathways[12, 48]. Although we have not seen these pathways previously identified in survivors with trait anxiety, anxiety and stress share similar biological underpinnings[2].

Preclinical models show that the PI3-Akt signaling pathway is upstream of the mTOR pathway and is implicated in increased anxiety behaviors when dysfunctional[49]. The p53 pathway, also shared between survivors and the general population, has previously been associated with increasing anxiety-like behaviors in preclinical models[50]. p53 is associated with behavioral changes that can cause the onset of GAD, since overactivation of the p53 tumor suppressor can further suppress the proliferation of neural stem cells and reinforce an anxious state in individuals[51]. Therefore, it is likely that this cell loss tied to the p53 signaling pathway, which leads to a hyperactivation of the stress response from the HPA axis, contributes to the pathogenesis of trait anxiety.

Interestingly, we found that pathways often associated with cancer targets such as MAPK and mTOR are not exclusive to cancer survivors, suggesting that transcriptional and cell-fate regulatory pathways form a general foundation for trait anxiety. The additional pathway perturbed in cancer survivors, prostate cancer, may be due to sex-specific differences in the patient population and warrants further investigation.

### Neurodegeneration & Neurotransmission

Neurodegeneration is a previously hypothesized mechanism contributing to anxiety[52–54], and higher trait anxiety is associated with neuroinflammation in patients with Alzheimer’s Disease[13], which is associated with neurodegeneration. Concordant with these hypotheses, the neuroactive ligand-receptor interaction pathway was perturbed in both cancer survivors and the non-cancer cohort in our study. Perturbations in this pathway are associated with cancer-related cognitive impairment in oncology patients[55] and are connected to clinical levels of anxiety in GAD[56]. Another common neurodegeneration pathway was Axon guidance. A clinical neuroimaging study of healthy women showed that increased strength of the axon pathway between the amygdala and prefrontal cortex predicted lower trait anxiety[57], suggesting that exploring the connectivity between the amygdala and its regulatory centers may provide predictive insight into trait anxiety.

The Alzheimer’s disease signaling pathway was common between the general population and cancer survivors. Trait anxiety is correlated with Alzheimer’s Disease pathogenesis, and this could be due to dysregulated activity of the HPA axis[58]. Notably, Parkinson’s disease and Huntington’s disease were uniquely perturbed in survivors. In a separate study of patients with cancer who self-reported cognitive changes and anxiety using the STAI, Parkinson’s Disease and Huntington’s Disease were also perturbed neurodegenerative pathways, pointing to a shared mechanism of these pathways in trait anxiety pathogenesis[15]. This could occur potentially through mitochondrial dysfunction, which was associated with anxiety-like behavior in a mouse model of Parkinson’s disease[15, 59].

Emerging evidence suggests that cancer and anti-cancer treatments may result in accelerated brain aging and neurodegenerative processes, perhaps reflecting distinct mechanisms within these processes as being related to trait anxiety among cancer survivors[60]. These findings suggest that neurodegeneration and trait anxiety are tightly linked. The further perturbations observed in survivors, including the olfactory transduction pathway, suggest a unique role for neurodegeneration in shaping trait anxiety within this population.

### Cell Structure & Regulation

Several pathways were related to cell structure and regulation, some of which have been previously associated with trait anxiety[11]. Shaping of the extracellular matrix (ECM) was previously identified as a mechanism contributing to trait anxiety[11], and the ECM-receptor interaction signaling pathway was perturbed in both the general population and cancer survivors. This pathway also reflects mechanisms of proliferation and migration, and genes involved in cellular transport were also found to be associated with measures of trait anxiety[11].

The endocytosis pathway was also common between the non-cancer cohort and cancer survivors. This may contribute to the pathogenesis of anxiety by regulating serotonin receptors and transporters[61]. Existing treatments for depression and anxiety, such as selective serotonin reuptake inhibitors (SSRIs), can improve their efficacy by modulating the endocytosis of serotonergic systems[61].

Cardiac muscle contraction was uniquely perturbed in the cancer survivor cohort. This direct link of cardiac function to trait anxiety in cancer survivors is a novel finding. Deviations in blood pressure and sympathetic nervous system activity are associated with trait anxiety[62], and alterations in cardiac function have been implicated in psychological stress and exercise responses in cancer patients[63, 64]. Furthermore, cardiotoxicity from cancer and its treatment, such as from chemotherapeutic agents, can contribute to physiological alterations of cardiac pathways and is associated with cardiotoxic properties such as myocardial injury, fluid retention, and arrythmia among others[65].

Regulation of the actin cytoskeleton was also distinctly perturbed in cancer survivors. Actin dynamics have been linked to shaping stress responses and complex behavior, including anxiety[66, 67]. In cancer survivors, specifically, the neurotoxicity introduced by chemotherapeutic drugs can induce cytoskeletal rearrangements, including abnormal rearrangements of actin microfilaments[68, 69]. Although this finding has been linked to peripheral neuropathy[69] and has not yet been tied directly to trait anxiety, it yields insight into why this pathway might be distinctly perturbed in cancer survivors.

Both progesterone-mediated oocyte maturation and oocyte meiosis were perturbed in both the non-cancer cohort and cancer survivors. These pathways have been linked to stress in females[70] and could influence trait anxiety through modulations of the HPA axis, central in stress physiology.

### Limitations and Future Directions

This study assessed pathway perturbations associated with trait anxiety severity in cancer survivors and the general population, using the MESA dataset as our patient population. While this study had well-phenotyped patients sampled from numerous diverse locations and populations, and set strict criteria for pathway perturbation selection, some limitations warrant consideration. Given the sample size of cancer survivors (n=74), further studies are needed to validate these results, since this evaluation is the first to report on associations between trait anxiety and gene expression changes. The MESA dataset did not include data on cancer survivors’ treatment regimens or time from diagnosis, factors that might influence how trait anxiety presents in survivors. While several characteristics were assessed in the MESA dataset, certain chronic, daily stressors such as financial precarity were not captured and should be evaluated in future research. In this study, we considered treatment with tricyclic anti-depressants. Future research should consider a more diverse range of anxiety treatments including monoamine oxidase inhibitors (MAOIs) and non-tricyclic antidepressants, which may also have anxiolytic effects[71]. Lastly, the MESA study was conducted in the United States and may require some degree of independent validation to be more generalizable.

## Conclusion

This study is the first to evaluate transcriptomic perturbations in signaling pathways associated with trait anxiety severity in cancer survivors and a matched non-cancer cohort. Findings from this study suggest that trait anxiety in cancer survivors is associated with common and distinct molecular mechanisms as compared to the general population. Pathways distinct to the survivor cohort included those related to neurodegeneration, cytoskeleton regulation, and cardiac function, suggesting that systemic dysregulations by cancer and/or its treatment may contribute to trait anxiety in cancer survivors. Focusing on these distinct pathways may inform the development of more targeted, precise therapeutics for trait anxiety in cancer survivors, while pathways shared with the general population provide insight into broader mechanisms of trait anxiety.

## Supplementary Material

Supplementary Files

This is a list of supplementary files associated with this preprint. Click to download.

- SupplementalFile1PIAresultsSurvivors.xlsx
- SupplementalFile2PIAresultsPSmatchedcohort.xlsx
- SupplementalTable1MESASTAIQuestions.docx
- SupplementalTable2SociodemographicPropensityScoreMatch.docx
- SupplementalTable4ComorbidityandOtherPSMMatched.docx
- SupplementalTable3ComorbidityandOtherTxSurvivors.docx
- SupplementalTable5PIAResultsInfectionDisease.docx

## Figures and Tables

**Table 1:** Differences in demographic and clinical characteristics in cancer survivors with low and high trait anxiety.

Characteristic	Low Trait Anxiety n = 63	High Trait Anxiety n = 11	Statistic
	Median (IQR)	Median (IQR)	
Age	69 (10)	66(13)	p = 0.46, t = 0.81
	% (n)	% (n)	
Gender			
female	43 (27)	82 (9)	p = 0.02, OR = 0.17
male	57 (36)	18 (2)	
Household income			
<$29,999	33 (21)	27 (3)	p = 0.65, *x*^2^=1.71
$30,000 – $74,999	37 (23)	55 (6)	
$75,000 – $100,000	6 (4)	0 (0)	
>$100,000	24 (15)	18 (2)	
Race/Ethnicity			
White, Caucasian	68 (43)	82 (9)	p = 0.82, *x*^2^=0.93
Chinese American	2 (1)	0 (0)	
Black, African-American	17 (11)	9 (1)	
Hispanic	13 (8)	9 (1)	
Employment			
Not working	49 (31)	45 (5)	p = 1.00, *x*^2^=0.05
Working for pay	51 (32)	55 (6)	
BMI categories			
Normal	29 (18)	36 (4)	p = 0.69, *x*^2^=1.25
Grade 1 Overweight	48 (30)	55 (6)	
Grade 2 Overweight	22 (14)	9 (1)	
Grade 3 Overweight	2 (1)	0 (0)	
Education			
High school or less	6 (4)	9 (1)	p = 0.31, *x*^2^=2.68
Some college, technical, or associate’s degree	44 (28)	18 (2)	
Bachelor’s degree or more	49 (31)	73 (8)	
Marital status			
Married	68 (43)	45 (5)	p = 0.17, *x*^2^=3.54
Widowed/divorced/separated	27 (17)	36 (4)	
Never married	5 (3)	18 (2)	
Cancer Diagnosis			
Breast only	13 (8)	9 (1)	p = 0.73, *x*^2^=2.44
Colon only	8 (5)	0 (0)	
NM Skin only	27 (17)	36 (4)	
Prostate only	8 (5)	18 (2)	
Other, multiple, or Not Specified	44 (28)	36 (4)	

**Table 2:** Differences in demographic and clinical characteristics in the propensity score matched cohort with low and high trait anxiety.

Characteristic	Low Trait Anxiety n = 181	High Trait Anxiety n = 41	Statistic
	Median (IQR)	Median (IQR)	
Age	69 (11)	70 (8)	
	% (n)	% (n)	
Gender			
female	49 (89)	63 (26)	p = 0.12, OR = 0.547
male	51 (93)	37 (15)	
Household income			
<$29,999	31 (57)	37 (15)	p = 0.12, *x*^2^=5.81
$30,000 – $74,999	38 (69)	51 (21)	
$75,000 – $100,000	8 (14)	2 (1)	
>$100,000	23 (41)	10 (4)	
Race/Ethnicity			
White, Caucasian	67 (121)	68 (28)	p = 0.68, *x*^2^=1.48
Chinese American	2 (3)	0 (0)	
Black, African-American	19 (34)	15 (6)	
Hispanic	13 (23)	17 (7)	
Employment			
Not working	47 (85)	56 (23)	p = 0.33, *x*^2^=1.12
Working for pay	53 (96)	44 (18)	
BMI categories			
Normal	32 (58)	20 (8)	p = 0.13, *x*^2^=5.42
Grade 1 Overweight	42 (76)	61 (25)	
Grade 2 Overweight	24 (44)	20 (8)	
Grade 3 Overweight	2 (3)	0 (0)	
Education			
High school or less	6 (10)	15 (6)	p = 0.07, *x*^2^=5.21
Some college, technical, or associate’s degree	42 (76)	46 (19)	
Bachelor’s degree or more	52 (95)	39 (16)	
Marital status			
Married	60 (109)	51 (21)	p < 0.01, *x*^2^=11.6
Widowed/divorced/separated	34 (62)	27 (11)	
Never married	6 (10)	22 (9)	

**Table 3:** Signaling pathways perturbed in cancer survivors.

Pathway ID	Pathway Name	Pert	FDR	Overlap with PS-matched cohort
**Inflammation & Immune Signaling**				
hsa04060	Cytokine-cytokine receptor interaction	11.22	0.007	Common
hsa04062	Chemokine signaling pathway	4.88	0.011	Common
hsa04672	Intestinal immune network for IgA production	6.40	0.009	Common
hsa04380	Osteoclast differentiation	4.43	0.020	Common
hsa05330	Allograft rejection	3.54	0.020	Common
hsa05322	Systemic lupus erythematosus	9.33	0.007	Common
hsa05323	Rheumatoid arthritis	7.37	0.007	Common
**Cellular Growth, Cycle, & Proliferation**				
hsa04151	PI3K-Akt signaling pathway	5.46	0.007	Common
hsa04010	MAPK signaling pathway	6.97	0.009	Common
hsa04012	ErbB signaling pathway	8.79	0.009	Common
hsa04150	mTOR signaling pathway	4.32	0.017	Common
hsa04390	Hippo signaling pathway	3.79	0.020	Common
hsa04115	p53 signaling pathway	5.27	0.015	Common
hsa04110	Cell cycle	5.21	0.011	Common
hsa05200	Pathways in cancer	6.81	0.007	Common
hsa05203	Viral carcinogenesis	4.53	0.007	Common
hsa05202	Transcriptional misregulation in cancer	3.69	0.015	Common
hsa05215	Prostate cancer	4.62	0.015	**Distinct**
hsa05210	Colorectal cancer	3.42	0.024	Common
**Neurodegeneration & Neurotransmission**				
hsa04080	Neuroactive ligand-receptor interaction	7.14	0.007	Common
hsa04740	Olfactory transduction	5.32	0.017	**Distinct**
hsa04360	Axon guidance	4.25	0.019	Common
hsa05012	Parkinson’s disease	5.29	0.009	**Distinct**
hsa05010	Alzheimer’s disease	4.52	0.011	Common
hsa05016	Huntington’s disease	3.54	0.016	**Distinct**
**Cell Structure & Regulation**				
hsa04512	ECM-receptor interaction	7.68	0.007	Common
hsa04510	Focal adhesion	5.63	0.013	Common
hsa04810	Regulation of actin cytoskeleton	4.37	0.016	**Distinct**
hsa04144	Endocytosis	3.61	0.015	Common
hsa04912	GnRH signaling pathway	7.36	0.009	Common
hsa04114	Oocyte meiosis	3.70	0.020	Common
hsa04914	Progesterone-mediated oocyte maturation	4.39	0.020	Common
hsa04260	Cardiac muscle contraction	4.28	0.013	**Distinct**
hsa04950	Maturity onset diabetes of the young	3.87	0.024	Common

**Table 4:** Signaling pathways perturbed in non-cancer cohort.

Pathway ID	Pathway Name	Pert	FDR	Overlap with survivors
**Inflammation & Immune Signaling**				
hsa04060	Cytokine-cytokine receptor interaction	36.72	0.002	Common
hsa04062	Chemokine signaling pathway	30.45	0.002	Common
hsa05323	Rheumatoid arthritis	32.58	0.002	Common
hsa05322	Systemic lupus erythematosus	11.09	0.002	Common
hsa05330	Allograft rejection	7.18	0.006	Common
hsa04380	Osteoclast differentiation	27.51	0.002	Common
hsa04672	Intestinal immune network for IgA production	6.90	0.007	Common
hsa04621	NOD-like receptor signaling pathway	30.74	0.002	**Distinct**
hsa04064	NF-kappa B signaling pathway	29.48	0.002	**Distinct**
hsa04620	Toll-like receptor signaling pathway	27.26	0.002	**Distinct**
hsa04623	Cytosolic DNA-sensing pathway	25.76	0.002	**Distinct**
hsa04622	RIG-I-like receptor signaling pathway	12.53	0.002	**Distinct**
hsa04630	Jak-STAT signaling pathway	4.01	0.010	**Distinct**
hsa04660	T cell receptor signaling pathway	4.64	0.013	**Distinct**
hsa04612	Antigen processing and presentation	8.65	0.007	**Distinct**
hsa04650	Natural killer cell mediated cytotoxicity	4.03	0.020	**Distinct**
hsa04145	Phagosome	6.19	0.006	**Distinct**
hsa04610	Complement and coagulation cascades	4.45	0.010	**Distinct**
**Cellular Growth, Cycle, & Proliferation**				
hsa04151	PI3K-Akt signaling pathway	4.04	0.010	Common
hsa04010	MAPK signaling pathway	25.8	0.002	Common
hsa04012	ErbB signaling pathway	13.11	0.005	Common
hsa04150	mTOR signaling pathway	7.19	0.008	Common
hsa04390	Hippo signaling pathway	5.53	0.006	Common
hsa04115	p53 signaling pathway	8.52	0.002	Common
hsa04110	Cell cycle	8.35	0.003	Common
hsa05200	Pathways in cancer	8.43	0.003	Common
hsa05203	Viral carcinogenesis	16.17	0.002	Common
hsa05202	Transcriptional misregulation in cancer	14.44	0.002	Common
hsa05210	Colorectal cancer	4.38	0.020	Common
hsa04210	Apoptosis	25.10	0.002	**Distinct**
hsa04350	TGF-beta signaling pathway	7.75	0.002	**Distinct**
hsa05219	Bladder cancer	10.15	0.002	**Distinct**
hsa05222	Small cell lung cancer	3.79	0.021	**Distinct**
hsa04310	Wnt signaling pathway	3.47	0.021	**Distinct**
hsa04066	HIF-1 signaling pathway	4.02	0.020	**Distinct**
**Neurodegeneration & Neurotransmission**				
hsa04080	Neuroactive ligand-receptor interaction	4.41	0.008	Common
hsa05010	Alzheimer’s disease	19.89	0.002	Common
hsa05014	Amyotrophic lateral sclerosis (ALS)	6.31	0.010	**Distinct**
hsa05034	Alcoholism	7.41	0.003	**Distinct**
hsa05030	Cocaine addiction	10.46	0.005	**Distinct**
hsa05031	Amphetamine addiction	10.23	0.005	**Distinct**
hsa04721	Synaptic vesicle cycle	4.24	0.018	**Distinct**
**Cell Structure & Regulation**				
hsa04512	ECM-receptor interaction	12.13	0.002	Common
hsa04510	Focal adhesion	5.67	0.008	Common
hsa04144	Endocytosis	3.88	0.008	Common
hsa04360	Axon guidance	4.27	0.011	Common
hsa04912	GnRH signaling pathway	5.95	0.006	Common
hsa04114	Oocyte meiosis	3.88	0.021	Common
hsa04914	Progesterone-mediated oocyte maturation	4.78	0.011	Common
hsa04950	Maturity onset diabetes of the young	6.92	0.008	Common
hsa04940	Type I diabetes mellitus	24.96	0.002	**Distinct**
hsa04930	Type II diabetes mellitus	5.38	0.011	**Distinct**
hsa04920	Adipocytokine signaling pathway	9.63	0.005	**Distinct**
hsa05410	Hypertrophic cardiomyopathy (HCM)	7.53	0.007	**Distinct**
hsa05414	Dilated cardiomyopathy	6.68	0.007	**Distinct**
hsa04960	Aldosterone-regulated sodium reabsorption	3.46	0.024	**Distinct**
hsa04723	Retrograde endocannabinoid signaling	3.83	0.013	**Distinct**

Abbreviations: FDR, False discovery rate. Pert, Total Normalized Perturbation Score
